# Literary Notes

**Published:** 1899-02

**Authors:** 


### LITERARY NOTES.

---

THE *Archives Provinciales de Médicine*, under the editorship of Dr. Marcel Baudouin, made its first appearance in Paris, on January 1, 1899. It is modeled after the *Archives Provinciales de Chirurgie*, and is published monthly in fasicules of 64 to 80 pages with numerous illustrations and will be devoted exclusively to medical work, written in the French language. Under the editorship of Dr. Baudouin, one of the ablest of French medical editors, this new publication will be a success from the beginning.

---

IT IS with much pleasure that the JOURNAL announces the advent of *The Archives of Neurology and Psychopathology*, a handsome quarterly of 262 pages, published under the auspices of the New York State

Hospitals and the Pathological Institute, by permission of the state board in lunacy, under the presidency of Dr. P. M. Wise. The editors for the state hospitals are Drs. G. Alder Blumer, Charles W. Pilgrim and Selden H. Talcott; for the pathological institute, Drs. Ira Van Gieson, Boris Sides and Henderson B. Deady.

These archives are to supplant the state hospitals bulletins, which for the past two years have contained so many interesting and valuable contributions from the staffs of the different state hospitals. The *Archives* will contain studies on abnormal mental life and their neural concomitants, based on psychology, psychopathology, experimental physiology and pathology, cellular biology, pathological anatomy, comparative neurology, physiological chemistry, anthropology and bacteriology.

No. 1 contains a carefully prepared paper on nervous energy by Drs. Ira Van Gieson and Boris Sides, and a very able and interesting address on the Correlation of sciences in the investigation of nervous and mental diseases, by Dr. Ira Van Gieson.

The *Archives* will be published in one volume annually, issued in four numbers. The subscription price per volume is $3.00 (12 shillings, 12 marks or 15 francs.) The price of single numbers will vary with their contents. Subscriptions are payable to the Manager, Archives of Neurology and Psychopathology, State Hospitals Press, Utica, N. Y.

---

THE *Southern Medical Journal*, edited at LaGrange, N. C., and published at Kinston, in the same state, made its appearance in January. It is edited by Dr. J. W. P. Southwick, who says in his salutatory "that he hopes to have a larger subscription list—one that will be the cause of envy of journals much our superior in age." We sincerely trust that the editor's hopes may be realised.

---

THE *Kansas Medical Journal*, which has been published for the last ten years in Topeka, Kansas, has been discontinued and its former editor, Dr. W. E. McVey, will have editorial control of the *Medical Monograph*, a 150-page monthly, which will be published in the place of the *Kansas Medical Journal*.

---

MESSRS. LEA BROTHERS & CO., of Philadelphia, Pa., announce for publication in March, 1899, the first volume of *Progressive Medicine*, a new annual which will be issued in four handsome octavo, cloth bound and richly illustrated volumes of about 400 pages each. The several volumes will appear at intervals of three months. This new quarterly will be edited by Professor Hobart Amory Hare, M. D., with whom will be associated some of the foremost medical authorities of the present day. It will be a condensed summary of medical progress, and will possess the advantage of giving this information four times a year. The subscription price is $10. The publishers offer to send full descriptive circulars and sample pages to those applying for them.

